# Wm. B. Keen & Co.

**Published:** 1867-10

**Authors:** 


### *Wm. B. Keen & Co.,*

The great wholesale and retail booksellers of this city, offer to the public an almost unlimited variety of educational, literary, scientific, and professional books. Fair and honorable dealing marks all their intercourse with their customers. We commend them to the entire confidence of all our friends who are in the market for medical books.

